# Author Correction: Carbonate chemistry and carbon sequestration driven by inorganic carbon outwelling from mangroves and saltmarshes

**DOI:** 10.1038/s41467-024-48384-0

**Published:** 2024-05-21

**Authors:** Gloria M. S. Reithmaier, Alex Cabral, Anirban Akhand, Matthew J. Bogard, Alberto V. Borges, Steven Bouillon, David J. Burdige, Mitchel Call, Nengwang Chen, Xiaogang Chen, Luiz C. Cotovicz, Meagan J. Eagle, Erik Kristensen, Kevin D. Kroeger, Zeyang Lu, Damien T. Maher, J. Lucas Pérez-Lloréns, Raghab Ray, Pierre Taillardat, Joseph J. Tamborski, Rob C. Upstill-Goddard, Faming Wang, Zhaohui Aleck Wang, Kai Xiao, Yvonne Y. Y. Yau, Isaac R. Santos

**Affiliations:** 1 Department of Marine Sciences, University of Gothenburg, 41319 Gothenburg, Sweden; 2 Department of Ocean Science, Hong Kong University of Science and Technology, Kowloon, Hong Kong SAR China; 3 Coastal and Estuarine Environment Research Group, Port and Airport Research Institute, 3-1-1 Nagase, Yokosuka, 239-0826 Japan; 4 Department of Biological Sciences, University of Lethbridge, Lethbridge, AB Canada; 5 Chemical Oceanography Unit, University of Liège, 4000 Liège, Belgium; 6 Department of Earth and Environmental Sciences, KU Leuven, 3001 Leuven, Belgium; 7 Department of Ocean and Earth Sciences, Old Dominion University, Norfolk, VA 23529 USA; 8 Faculty of Science and Engineering, Southern Cross University, Lismore, NSW 2480 Australia; 9 State Key Laboratory of Marine Environment Science, Xiamen University, Xiamen, 361102 China; 10 School of Engineering, Westlake University, Hangzhou, 310024 China; 11 Department of Marine Chemistry, Leibniz Institute for Baltic Sea Research, Warnemünde, Germany; 12 Institute of Marine Sciences (LABOMAR), Federal University of Ceará (UFC), Fortaleza, Brazil; 13 Woods Hole Coastal and Marine Science Center, U.S. Geological Survey, 384 Woods Hole Road, Woods Hole, MA 02543 USA; 14 Department of Biology, University of Southern Denmark, 5230 Odense, Denmark; 15 College of the Environment and Ecology, Xiamen University, Xiamen, 361102 China; 16 Instituto Universitario de Investigación Marina (INMAR), University of Cádiz, Puerto Real, Cádiz, Spain; 17 Atmosphere and Ocean Research Institute, The University of Tokyo, Tokyo, Japan; 18 NUS Environmental Research Institute, National University of Singapore, Singapore, 117411 Singapore; 19 School of Natural and Environmental Sciences, Newcastle University, Newcastle upon Tyne, NE1 7RU UK; 20 Xiaoliang Research Station of Tropical Coastal Ecosystems, Chinese Academy of Sciences, Guangzhou, 510650 China; 21 Department of Marine Chemistry and Geochemistry, Woods Hole Oceanographic Institution, Woods Hole, MA 02543 USA; 22 School of Environmental Science and Engineering, Southern University of Science and Technology, Shenzhen, China

**Keywords:** Carbon cycle, Hydrology, Carbon cycle

Correction to: *Nature Communications* 10.1038/s41467-023-44037-w, published online 11 December 2023

The original version of the Supplementary Information associated with this Article cited Santos et al. (unpublished) for the sites Johnstone, Burdekin and Fitzroy in Supplementary Table 1. This has been changed to Rosentreter & Eyre (2024). The HTML has been updated to include a corrected version of the Supplementary Information.

