# Supplementary material for: Carbonate chemistry and carbon sequestration driven by inorganic carbon outwelling from mangroves and saltmarshes
Source: Nat Commun. 2023 Dec 11;14:8196. doi: 10.1038/s41467-023-44037-w (PMC10713528; doi:10.1038/s41467-023-44037-w)
Supplement: Supplementary file 1 — Supplementary Information [file 41467_2023_44037_MOESM1_ESM.pdf]

Supporting Information

Carbonate chemistry and carbon sequestration driven by inorganic carbon outwelling from mangroves and saltmarshes

Gloria M. S. Reithmaier, Alex Cabral, Anirban Akhand, Matthew J. Bogard, Alberto V. Borges, Steven Bouillon, David J. Burdige, Mitchel Call, Nengwang Chen, Xiaogang Chen, Luiz C. Cotovicz Jr, Meagan J. Eagle, Erik Kristensen, Kevin D. Kroeger, Zeyang Lu, Damien T. Maher, J. Lucas Pérez-Lloréns, Raghab Ray, Pierre Taillardat, Joseph J. Tamborski, Rob C. Upstill-Goddard, Faming Wang, Zhaohui Aleck Wang, Kai Xiao, Yvonne Y. Y. Yau, and Isaac R. Santos

Extended data

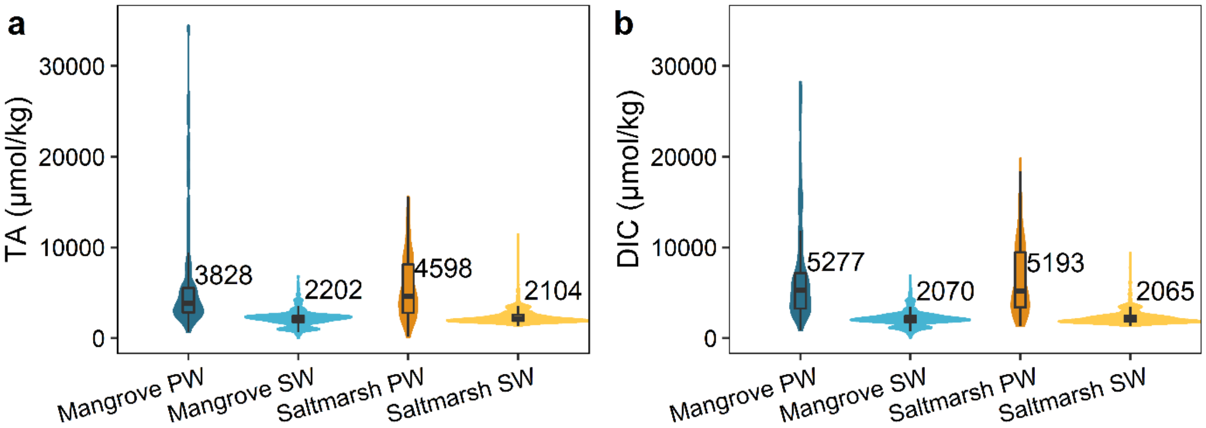

Fig. S1 | a, TA and (b) DIC concentrations in porewater (PW) were two- to three-times higher than concentrations in surface water (SW). Numbers show medians. Outliers were excluded.

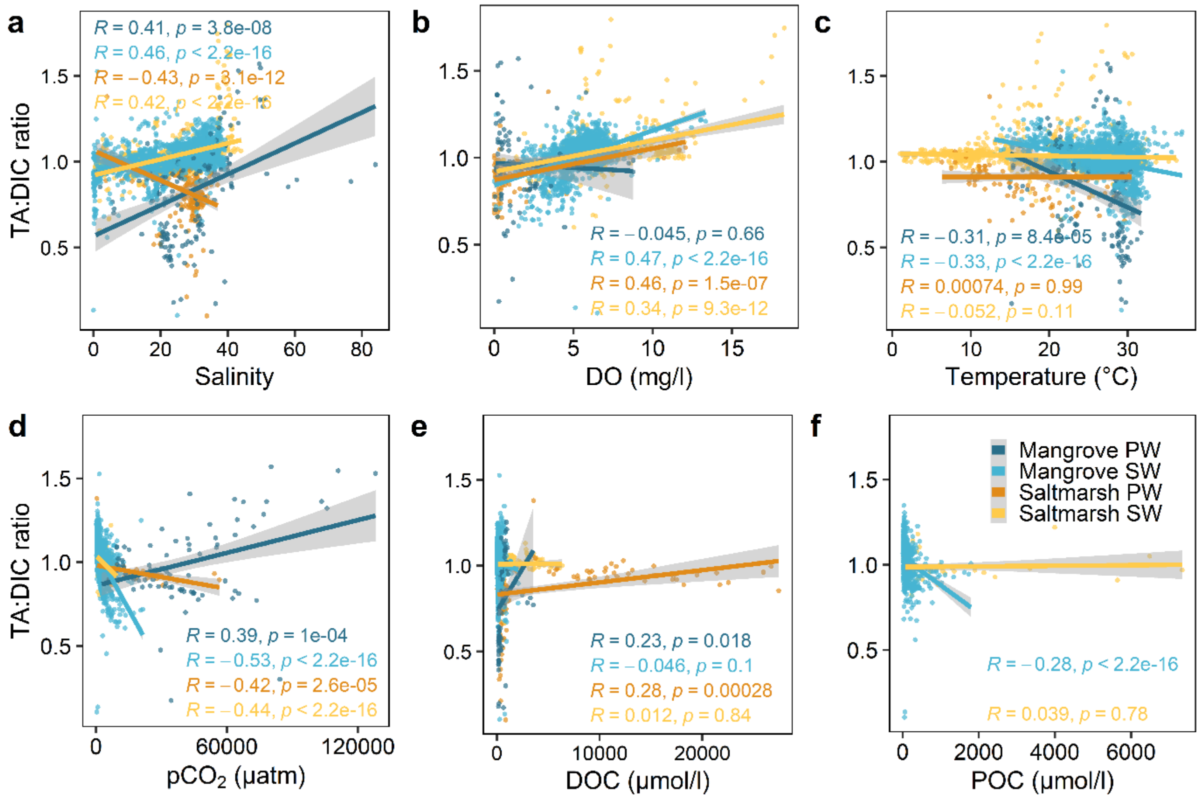

Fig. S2 | Potential drivers of TA:DIC ratios in intertidal wetlands. Correlations between TA:DIC ratios and (**a**) salinity, (**b**) dissolved oxygen, (**c**) temperature, (**d**) partial pressure of carbon dioxide, (**e**) dissolved organic carbon, and (**f**) particulate organic carbon in porewater (PW) and surface water (SW).

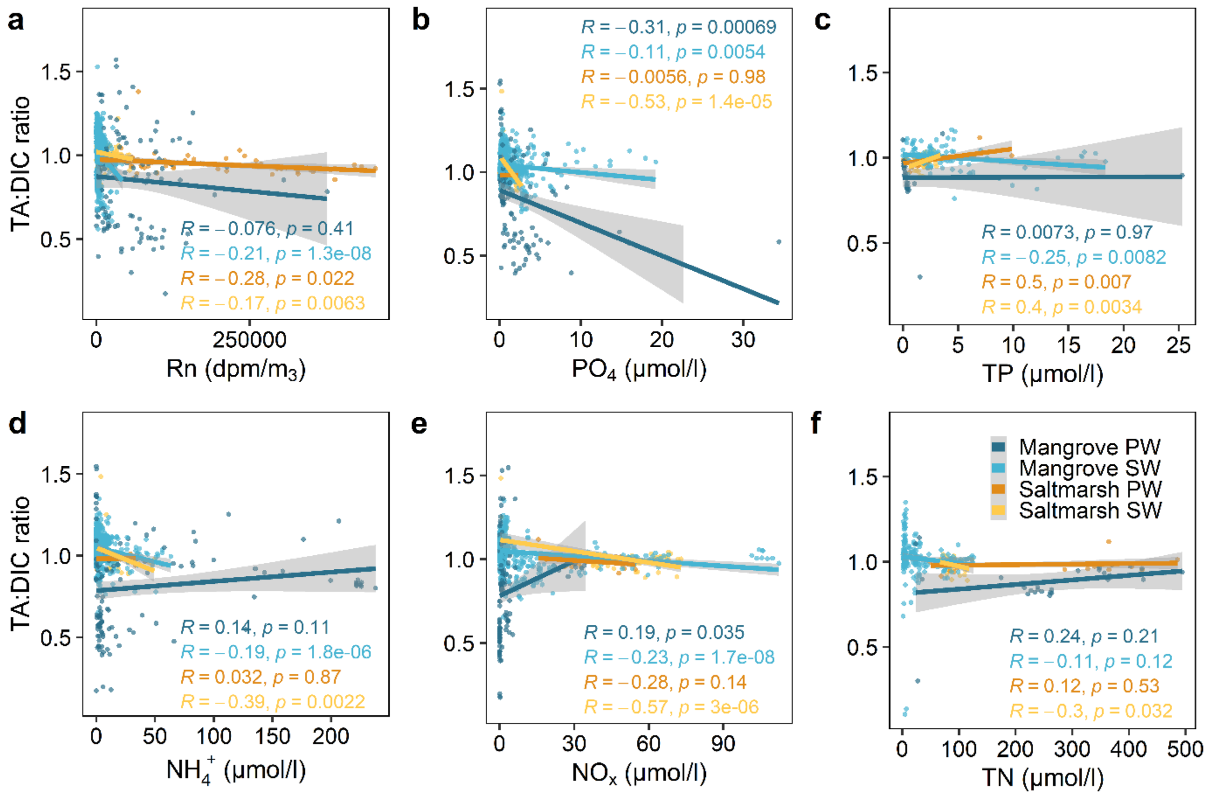

Fig. S3 | Potential drivers of TA:DIC ratios in intertidal wetlands. Correlations between TA:DIC ratios and (**a**) porewater tracer radon-222, (**b**) phosphate, (**c**) total phosphorus, (**d**) ammonium, (**e**) nitrogen oxides (nitrate and nitrite), and (**f**) total nitrogen in porewater (PW) and surface water (SW).

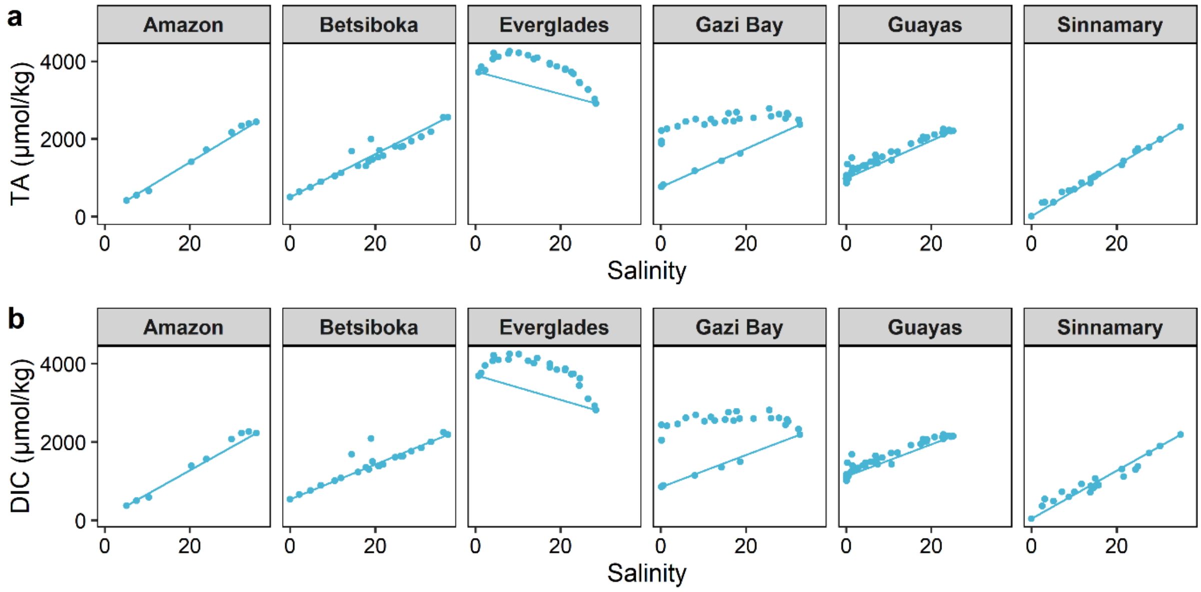

Fig. S4 | Pristine mangrove estuaries are sources for TA and DIC. a, TA and (**b**) DIC plotted against salinity, and conservative mixing lines, measured during spatial surveys. Conservative mixing lines were estimated from TA and DIC concentrations at the lowest and highest salinities at each site.

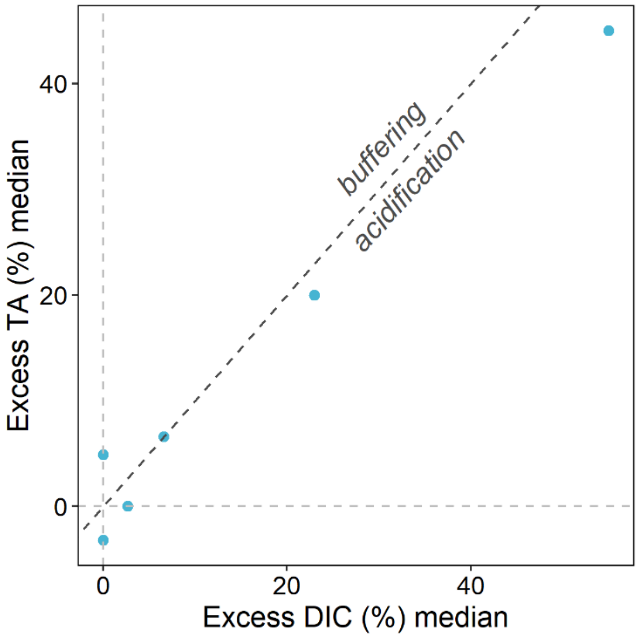

Fig. S5 | Four out of six pristine mangrove-dominated estuaries had larger estuarine DIC than TA inputs. The standard estuarine mixing model was used to calculate estuarine TA and DIC sources/sinks in surface water along six pristine mangrove estuaries with a clear salinity gradient. Deviations between TA and DIC concentrations, measured during spatial surveys, and conservative mixing lines were calculated as a percentage and averaged for each estuary. Positive excess values indicate a source within the estuary, whereas negative values indicate a sink.

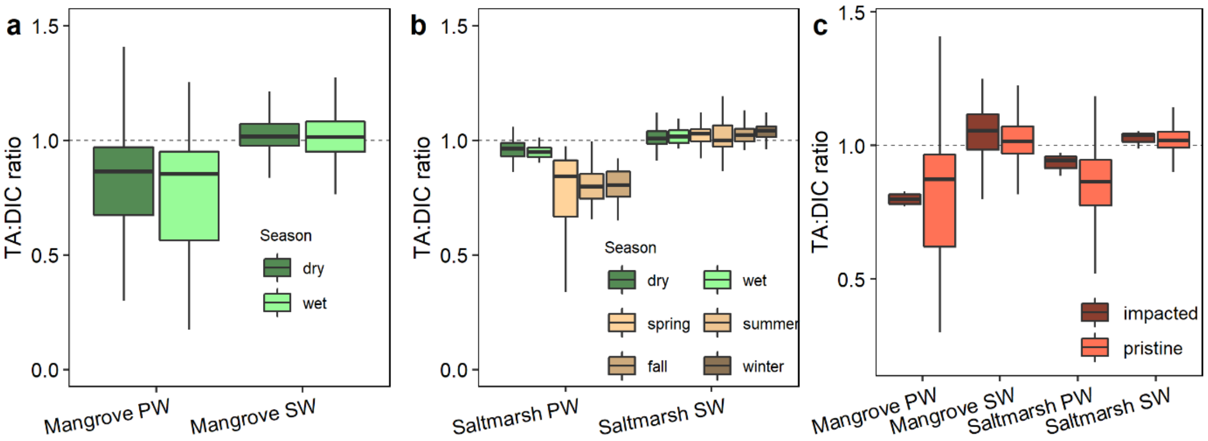

Fig. S6 | Seasonal changes of TA:DIC ratios in coastal wetlands and anthropogenic impacts. TA:DIC ratios measured during different seasons in porewater (PW) and surface water (SW) at (**a**) mangroves and (**b**) saltmarshes. **c**, TA:DIC ratios in pristine versus anthropogenically impacted sites. Outliers were excluded from the graph.

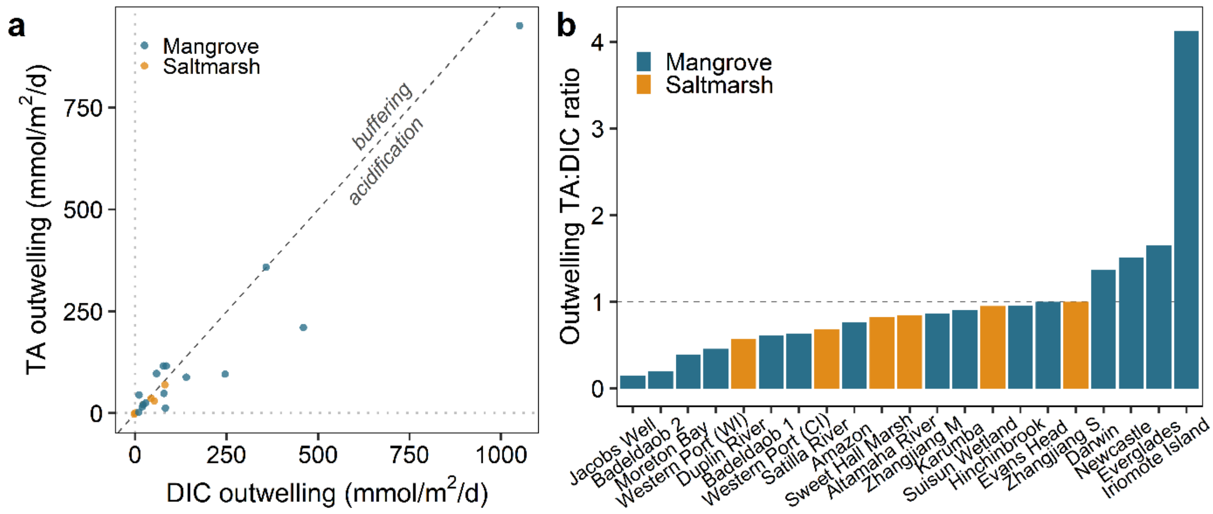

Fig. S7 | Most intertidal wetlands had higher DIC than TA outwelling rates. **a**, Regression between TA and DIC outwelling and (**b**) TA:DIC outwelling ratios per site.

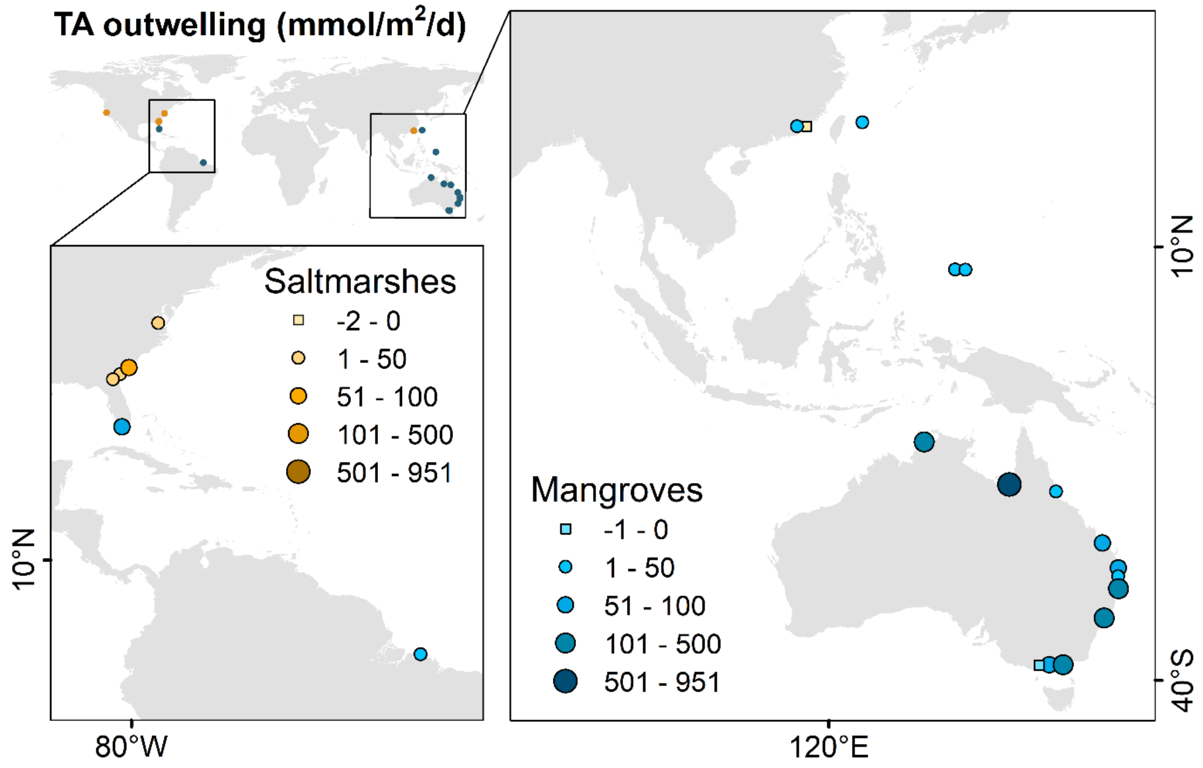

Fig. S8 | Most studies measuring TA outwelling rates from intertidal wetlands were conducted in the USA and Australia. Rates are scaled to the interintertidal wetland area. The location of some sites was adjusted slightly to allow visualization. The precise coordinates are available on Table S3.

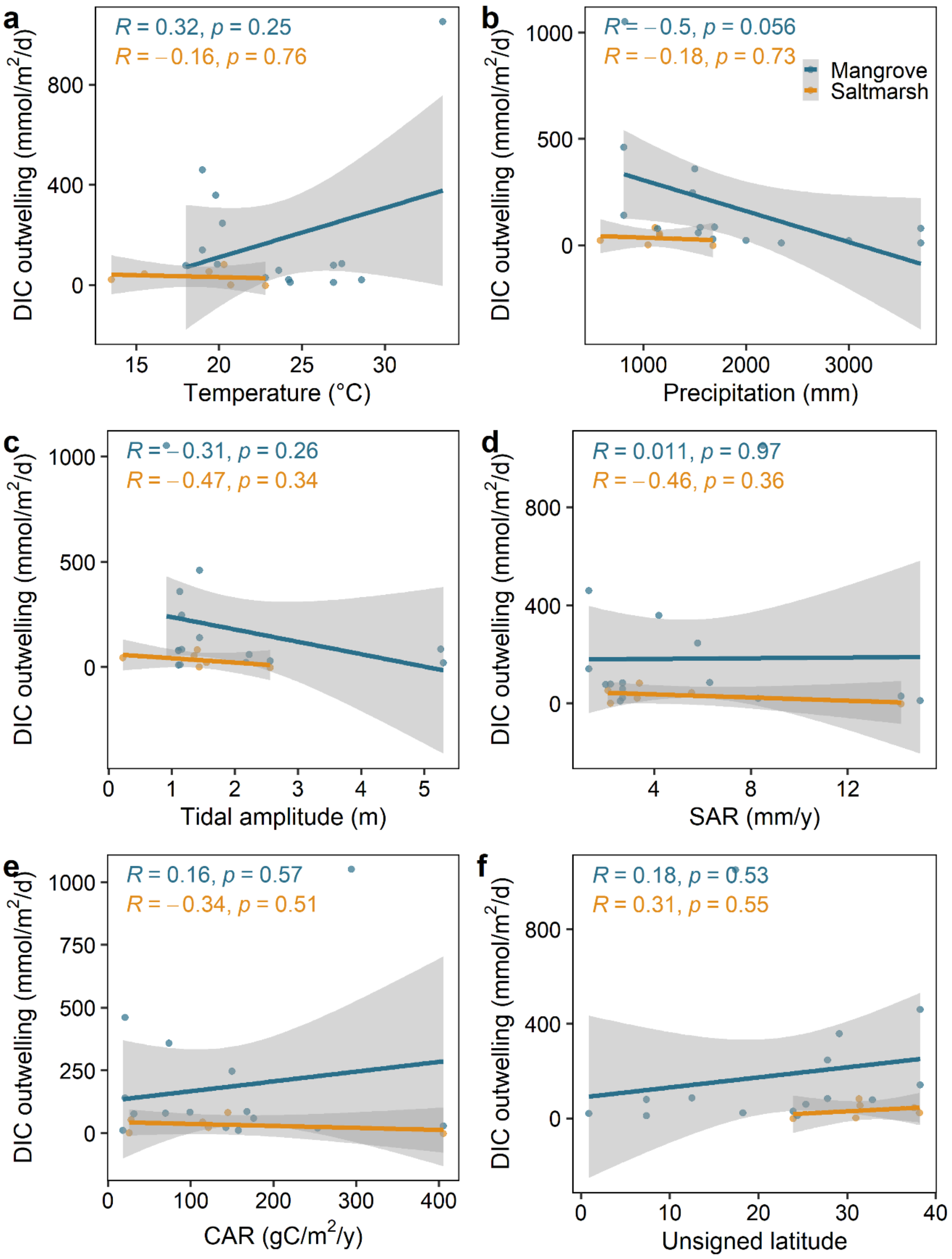

Fig. S9 | Potential drivers of DIC outwelling in intertidal wetlands. Regressions between TA:DIC outwelling ratios and (**a**) average annual temperature, (**b**) average annual precipitation, (**c**) intertidal amplitude, (**d**) sediment accumulation rates, (**e**) carbon accumulation rates, and (**f**) unsigned latitudes.

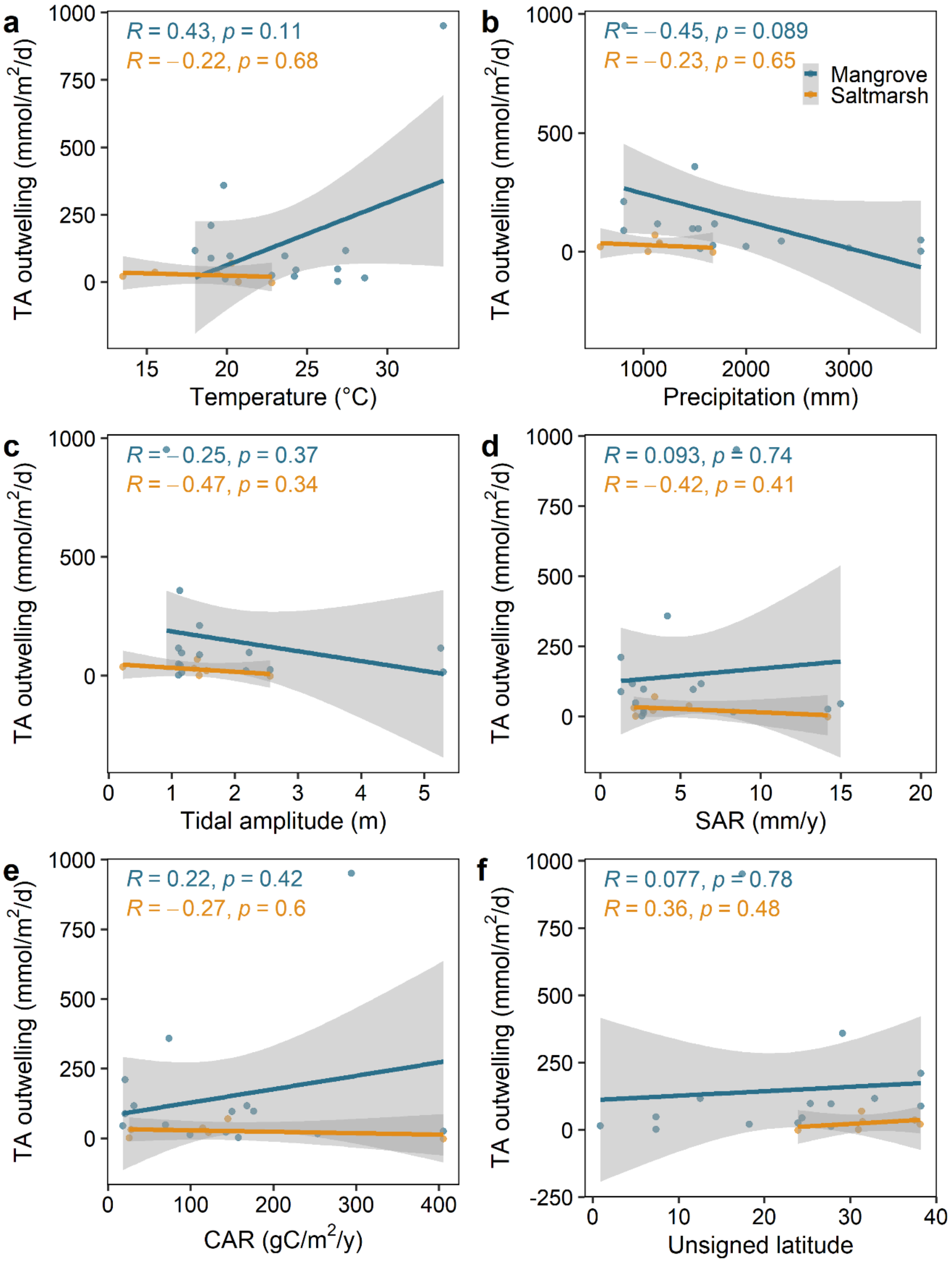

Fig. S10 | Potential drivers of TA outwelling in intertidal wetlands. Regressions between TA:DIC outwelling ratios and (**a**) average annual temperature, (**b**) average annual precipitation, (**c**) intertidal amplitude, (**d**) sediment accumulation rates, (**e**) carbon accumulation rates, and (**f**) unsigned latitudes.

Use of TA:DIC ratios

The ratio of CO_3^{2-} to HCO_3^- is a major property of carbonate chemistry and determines the buffering capacity of seawater. This is reflected in the Revelle factor (and other buffer factors) that depends on the ratio of CO_3^{2-} to HCO_3^-, which is proportional to TA:DIC (Egleston, et al. [1]). Consequently, TA:DIC ratios drive pH changes and influence the capacity of seawater to take up anthropogenic CO_2, affecting ocean acidification and carbon sequestration.

The change in pH with increasing TA is relatively minor (e.g., pH increases from 7.41 to 7.48 when TA increases from 2000 to 10000 µmol/kg at a fixed TA:DIC = 1) compared to the changes in pH associated with changing TA:DIC ratios (e.g., pH increases from 6.50 to 7.41 when TA:DIC ratio increases from 0.8 to 1 when DIC = 2000 µmol/kg, Figure S1 and 2).

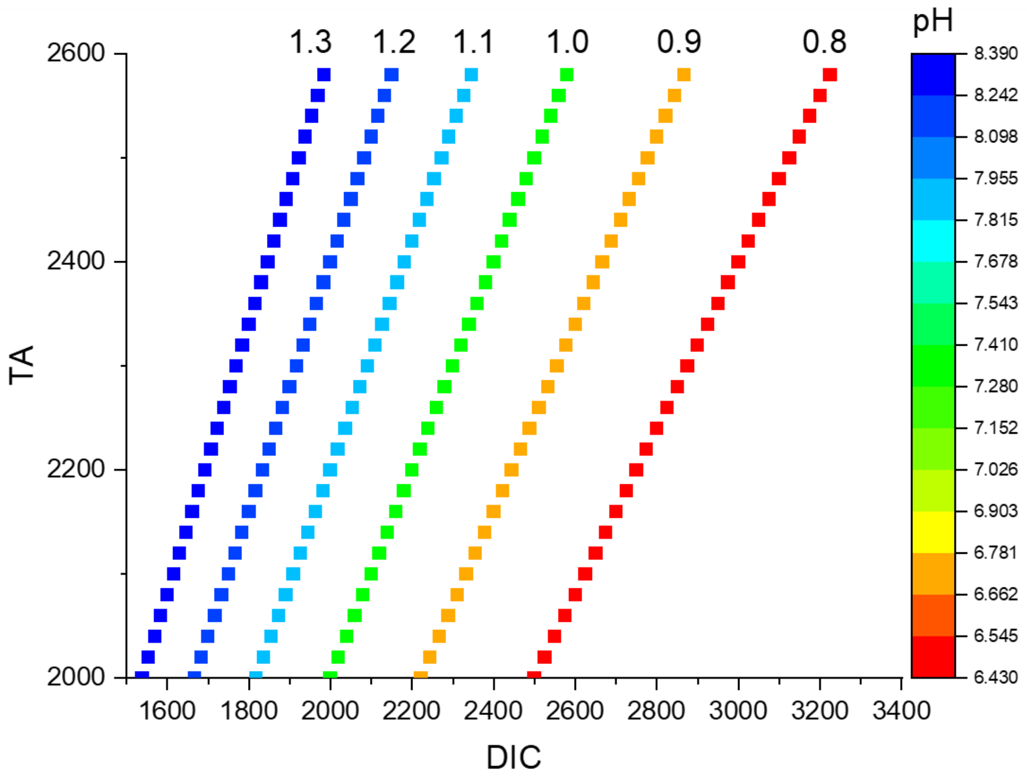

Fig. S11. Increasing TA:DIC ratios result in increasing pH.

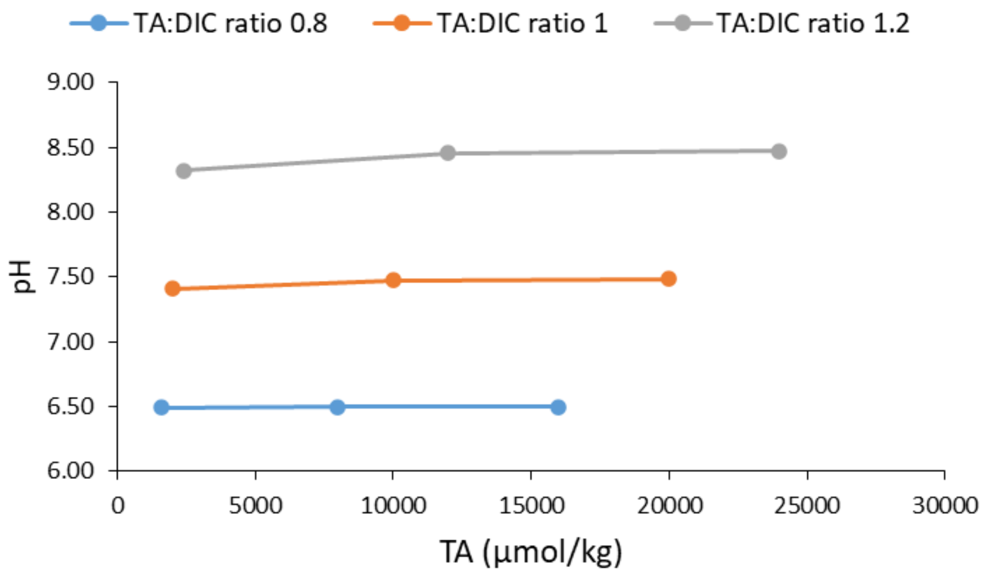

Fig. S12. pH as a function of increasing alkalinity at fixed TA:DIC ratios.

The linear regressions of TA:DIC ratios show a significantly positive trend using either pH or H^+ concentrations (Figure 2 and Figure S3). The scatter around TA:DIC ~ 1 is due to the minimum buffer capacity at this point, where a given increase in CO_2 will cause a larger decrease in pH compared to when TA:DIC > 1 (Wang, et al. [2]).

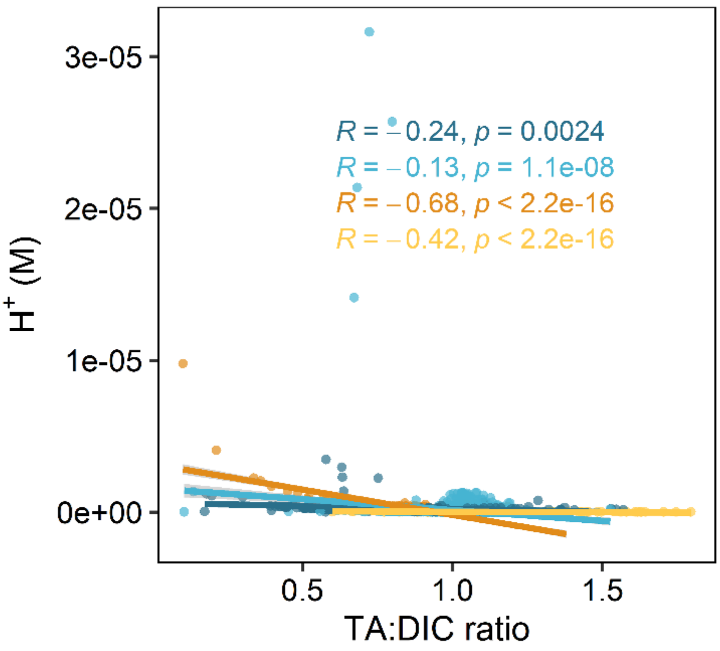

Fig. S13. Regressions between H^+ and TA:DIC ratios.

Table S1 | Information about sites with TA and DIC observations, including site ID, location, sampling type, i.e., and in porewater (PW) or in surface water measured during time series (TS) or spatial surveys (SV), number of samples, study length, season, and reference. Total sample numbers were 171 in mangrove porewaters, 1945 in mangrove surface waters, 243 in saltmarsh porewaters, and 940 in saltmarsh surface waters.

Ecosystem	Country	ID	Site	Condition	Latitude	Longitude	Type	Sample number	Study length (d)	Season	Reference
Saltmarsh	MA, USA	S01	Sage Lot Pond	Pristine creek	41.555	-70.5071	TS	291	35	annual	Tamborski, et al. [3]<br>Wang, et al. [2]<br>Song, et al. [4]<br>Chu, et al. [5]
Saltmarsh	MA, USA	S01	Sage Lot Pond	Pristine creek	41.555	-70.507	PW	145	11	annual	Brooks, et al. [6]<br>Tamborski, et al. [3]
Saltmarsh	CA, USA	S02	Suisun Marsh	Pristine creek	38.195	-122.033	TS	31	2	annual	Bogard, et al. [7]
Saltmarsh	SC, USA	S03	Oyster Landing	Pristine creek	33.333	-79.200	TS	13	1	summer	Correa, et al. [8]
Saltmarsh	SC, USA	S03	Oyster Landing	Pristine creek	33.333	-79.200	PW	8	3	summer	Correa, et al. [8]
Saltmarsh	GA, USA	S04	Duplin River	Pristine creek	31.421	-81.296	TS	223	19	annual	Wang and Cai [9]
Saltmarsh	Spain	S05	Los Toruños	Pristine creek	36.561	-6.207	TS	89	10	annual	Pérez-Lloréns, et al. [10]<br>Chen, et al. [12]<br>Yau, et al. [11]
Saltmarsh	China	S06	Chuandong	Pristine creek	33.047	120.867	TS	153	2	dry	Yau, et al. [11]<br>Chen, et al. [12]
Saltmarsh	China	S06	Chuandong	Pristine creek	33.047	120.867	PW	54	11	dry	Yau, et al. [11]<br>Chen, et al. (unpublished)
Saltmarsh	China	S06	Chuandong	Pristine creek	33.037	120.841	SV	53	2	dry	Yau, et al. [11]<br>Chen, et al. (unpublished)
Saltmarsh	China	S07	Hangzhou Bay	Impacted creek	30.355	121.128	SV	36	5	spring	Zhu, et al. [13]
Saltmarsh	China	S07	Hangzhou Bay	Impacted creek	30.355	121.128	PW	8	4	spring	Zhu, et al. [13]
Saltmarsh	China	S08	Zhangjiang E.	Impacted creek	23.921	117.426	TS	51	2	dry	Lu, et al. (unpublished)
Saltmarsh	China	S08	Zhangjiang E.	Impacted creek	23.921	117.426	PW	28	4	dry	Lu, et al. (unpublished)
Mangrove	FL, USA	M01	Everglades	Pristine estuary	25.362	-81.085	TS	57	2	dry	Reithmaier, et al. [14]
Mangrove	FL, USA	M01	Everglades	Pristine estuary	25.362	-81.085	SV	24	1	dry	Reithmaier, et al. [14]

Mangrove	Japan	M02	Iriomote	Pristine estuary	24.384	123.887	TS	6	3	wet	Akhand, et al. [15]
Mangrove	China	M03	Zhangjiang E.	Impacted creek	23.925	117.422	TS	52	2	dry	Lu, et al. (unpublished)
Mangrove	China	M03	Zhangjiang E.	Impacted creek	23.925	117.422	PW	25	4	dry	Lu, et al. (unpublished)
Mangrove	India	M04	Sundarbans	Pristine estuary and creeks	22.002	88.722	TS	113		annual	Ray, et al. [16]<br>Akhand, et al. [17]<br>Akhand, et al. [18]<br>Akhand, et al. [19]<br>Akhand, et al. [20]
Mangrove	India	M05	Bhitarkanika	Pristine estuary	20.777	86.847	TS	43	16	annual	Akhand, et al. [20]
Mangrove	India	M06	Gaderu	Pristine creek	16.869	82.285	SV	11	3	dry	Bouillon, et al. [21]
Mangrove	India	M06	Gaderu	Pristine creek	16.793	82.304	TS	25	1	dry	Borges, et al. [22]
Mangrove	India	M07	Kalighat	Pristine creek	13.127	92.947	TS	48	2	dry	Linto, et al. [23]
Mangrove	India	M07	Kalighat	Pristine creek	13.118	92.944	SV	30	2	dry	Linto, et al. [23]
Mangrove	India	M08	Wright Myo	Pristine creek	11.971	92.707	TS	96	4	dry	Linto, et al. [23]
Mangrove	India	M08	Wright Myo	Pristine creek	11.821	92.676	SV	60	4	dry	Linto, et al. [23]
Mangrove	India	M09	Kochi	Impacted creek	9.924	76.322	TS	23	1	dry	Santos, et al. (unpublished)
Mangrove	Philippines	M10	Panay	Pristine creek	11.806	122.202	TS	34	2	annual	Ray, et al. [24]
Mangrove	Philippines	M10	Panay	Pristine creek	11.806	122.202	SV	23	1	annual	Ray, et al. [24]
Mangrove	Vietnam	M11	Can Gio	Pristine creek	10.506	106.883	TS	162	7	annual	Taillardat, et al. [25]<br>Taillardat, et al. [26]
Mangrove	Vietnam	M11	Can Gio	Pristine creek	10.506	106.883	PW	40	10	annual	Taillardat, et al. [25]<br>Taillardat, et al. [26]
Mangrove	Vietnam	M12	Ho Cooc	Impacted creek	9.813	106.608	SV	12	4	annual	Borges, et al. [27]
Mangrove	Vietnam	M13	Ca Mau	Pristine creek	8.716	104.965	SV	63	8	annual	Borges, et al. [27]
Mangrove	Thailand	M14	Bangrong	Pristine creek	8.050	98.416	TS	45	2	annual	Kristensen, et al. (unpublished)
Mangrove	Palau	M15	Badeldaob 2	pristine creek	7.391	134.586	TS	28	1	wet	Call, et al. [28]
Mangrove	Palau	M15	Badeldaob 2	pristine creek	7.391	134.586	PW	3	2	wet	Call, et al. [28]
Mangrove	Palau	M16	Badeldaob 1	pristine creek	7.367	134.578	TS	28	1	wet	Call, et al. [28]
Mangrove	Palau	M16	Badeldaob 1	pristine creek	7.367	134.578	PW	3	2	wet	Call, et al. [28]
Mangrove	French Guiana	M17	Sinnamary	Pristine estuary	5.450	-53.011	TS	20	1	dry	Ray, et al. [29]
Mangrove	French Guiana	M17	Sinnamary	Pristine estuary	5.450	-53.011	SV	19		dry	Ray, et al. [30]
Mangrove	Brazil	M18	Amazon	Pristine estuary	-0.878	-46.629	SV	9	1	dry	Cabral, et al. [31]

Mangrove	Brazil	M18	Amazon	Pristine creek	-0.878	-46.629	TS	40	8	dry	Cabral, et al. [31]
Mangrove	Brazil	M18	Amazon	Pristine creek	-0.925	-46.618	PW	4	1	dry	Cabral, et al. [31]
Mangrove	Brazil	M19	Paraiba	Pristine creek	-21.604	-41.052	TS	12	1	wet	Cotovicz Jr, et al. [32]
Mangrove	Brazil	M20	Paraty	Pristine creek	-23.302	-44.649	TS	57	2	wet	Cabral, et al. (unpublished)
Mangrove	Brazil	M20	Paraty	Pristine creek	-23.302	-44.649	PW	12	2	wet	Cabral, et al. (unpublished)
Mangrove	Brazil	M21	Florianopolis	Pristine creek	-27.649	-48.553	TS	61	2	dry	Cabral, et al. (unpublished)
Mangrove	Brazil	M21	Florianopolis	Pristine creek	-27.649	-48.553	PW	12	2	dry	Cabral, et al. (unpublished)
Mangrove	Ecuador	M22	Guayas	Pristine estuary	-2.370	-79.842	SV	49	11	annual	Belliard, et al. [33]
Mangrove	Ecuador	M22	Guayas	Pristine estuary	-2.506	-79.874	TS	54	2	annual	Belliard, et al. [33]
Mangrove	Kenya	M23	Tana	Pristine estuary and creeks	-2.540	40.536	SV	52	10	wet	Bouillon, et al. [34]
Mangrove	Kenya	M23	Tana	Pristine estuary and creeks	-2.540	40.536	PW	8	5	wet	Bouillon, et al. [34]
Mangrove	Kenya	M24	Gazi Bay	Pristine estuary and creeks	-4.413	39.511	SV	46	11	dry	Bouillon, et al. [35]
Mangrove	Papua New Guinea	M25	Nagada	Pristine creek	-5.150	145.800	TS	39	5	dry	Borges, et al. [22]
Mangrove	Tanzania	M26	Ras Dege	Pristine creek	-6.876	39.457	TS	23	1	dry	Bouillon, et al. [36]
Mangrove	Tanzania	M26	Ras Dege	Pristine creek	-6.876	39.457	SV	22	4	annual	Bouillon, et al. [36]
Mangrove	Tanzania	M27	Mtoni	Impacted creek	-6.880	39.308	TS	24	1	dry	Bouillon, et al. [36]
Mangrove	Tanzania	M27	Mtoni	Impacted creek	-6.880	39.308	SV	18	4	annual	Bouillon, et al. [36]
Mangrove	Madagascar	M28	Betsiboka	Pristine estuary	-15.888	46.328	SV	23	6	wet	Ralison, et al. [37]
Mangrove	Australia	M29	Darwin	Pristine creek	-12.442	130.871	PW	12	2	dry	Sippo, et al. [38]
Mangrove	Australia	M29	Darwin	Pristine creek	-12.520	130.906	TS	24	1	dry	Sippo, et al. [38]
Mangrove	Australia	M30	Johnstone	Pristine creek	-17.529	146.061	TS	26	1	dry	Rosentreter and Eyre [39]
Mangrove	Australia	M31	Hinchinbrook	Pristine creek	-18.244	146.228	TS	24	1	dry	Sippo, et al. [38]
Mangrove	Australia	M31	Hinchinbrook	Pristine creek	-18.256	146.266	PW	12	2	dry	Sippo, et al. [38]
Mangrove	Australia	M32	Burdekin	Pristine creek	-19.623	147.565	TS	27	1	dry	Rosentreter and Eyre [39]
Mangrove	Australia	M33	Fitzroy	Pristine creek	-23.513	150.785	TS	26	1	dry	Rosentreter and Eyre [39]
Mangrove	Australia	M34	1770	Pristine creek	-24.189	151.879	PW	6	2	dry	Sippo, et al. [38]
Mangrove	Australia	M34	1770	Pristine creek	-24.192	151.570	TS	25	1	dry	Sippo, et al. [38]
Mangrove	Australia	M35	Jacobs Well	Pristine creek	-27.780	153.381	PW	9	2	annual	Sippo, et al. [38]
Mangrove	Australia	M35	Jacobs Well	Pristine creek	-27.781	153.380	TS	83	3	annual	Sippo, et al. [38]

Mangrove	Australia	M36	Evans Head	Pristine creek	-29.121	153.428	TS	108	4	annual	Santos, et al. [40]
Mangrove	Australia	M36	Evans Head	Pristine creek	-29.121	153.428	PW	4	1	dry	Santos, et al. [40]
Mangrove	Australia	M37	Newcastle	Pristine creek	-32.850	151.768	PW	12	2	wet	Sippo, et al. [38]
Mangrove	Australia	M37	Newcastle	Pristine creek	-32.851	151.768	TS	25	1	wet	Sippo, et al. [38]
Mangrove	Australia	M38	Barwon Heads	Pristine creek	-38.257	144.487	TS	25	1	wet	Sippo, et al. [38]
Mangrove	Australia	M38	Barwon Heads	Pristine creek	-38.264	144.497	PW	9	2	wet	Sippo, et al. [38]

Table S2 | TA:DIC ratios, TA:DIC slopes, TA_n:DIC_n slopes per site in porewater (PW) and surface water (SW), and tidal range during study periods.

Ecosystem	ID	Site	Type	TA:DIC ratio (median)	TA:DIC ratio (min)	TA:DIC ratio (max)	TA:DIC slope	TA:DIC slope R^2	TA_n:DIC_n slope	TA_n:DIC_n slope R^2	Tidal range (m)
Saltmarsh	S01	Sage Lot Pond	PW	0.796	0.102	1.18	0.82	0.96	0.82	0.95	NA
Saltmarsh	S01	Sage Lot Pond	SW	1.04	0.885	1.24	0.67	0.71	0.61	0.6	0.975
Saltmarsh	S02	Suisun Marsh	SW	0.972	0.6	1	0.94	0.65	0.97	0.83	1.62
Saltmarsh	S03	Oyster Landing	PW	0.88	0.656	0.937	0.73	0.83	0.79	0.86	NA
Saltmarsh	S03	Oyster Landing	SW	0.948	0.802	1.06	1.07	0.45	0.86	0.98	1.27
Saltmarsh	S04	Duplin River	SW	1.01	0.932	1.09	0.92	0.93	0.61	0.84	6.68
Saltmarsh	S05	Los Toruños	SW	1.14	0.996	1.8	0.58	0.48	0.59	0.5	NA
Saltmarsh	S06	Chuandong	PW	0.942	0.855	1.38	0.89	0.99	0.89	0.99	NA
Saltmarsh	S06	Chuandong	SW	1.02	0.822	1.22	0.98	0.97	1	0.97	4.12
Saltmarsh	S07	Hangzhou Bay	PW	0.943	0.886	0.974	0.89	0.99	0.9	0.99	NA
Saltmarsh	S07	Hangzhou Bay	SW	1.04	0.904	1.05	1	0.99	1	0.99	NA
Saltmarsh	S08	Zhangjiang E.	PW	0.982	0.916	1.12	0.88	0.96	0.9	0.96	NA
Saltmarsh	S08	Zhangjiang E.	SW	0.991	0.891	1.04	0.74	0.94	0.72	0.94	3.26
Mangrove	M01	Everglades	SW	1	0.948	1.11	0.94	0.97	0.92	0.92	0.668
Mangrove	M02	Iriomote	SW	1.16	1.02	1.18	0.79	0.64	0.68	0.72	NA
Mangrove	M03	Zhangjiang E.	PW	0.896	0.802	0.982	0.77	0.99	0.76	0.96	NA
Mangrove	M03	Zhangjiang E.	SW	0.998	0.904	1.1	0.97	0.82	0.97	0.82	3.26
Mangrove	M04	Sundarbans	SW	1.08	0.106	1.11	0.98	0.45	0.83	0.34	NA
Mangrove	M05	Bhitarkanika	SW	1.02	0.988	1.1	1.06	0.99	1.01	0.97	NA
Mangrove	M06	Gaderu	SW	1.03	0.976	1.2	0.63	0.83	0.61	0.97	1.37
Mangrove	M07	Kalighat	SW	1.01	0.914	1.21	0.76	0.82	0.92	0.94	0.255
Mangrove	M08	Wright Myo	SW	0.989	0.454	1.16	0.83	0.85	0.62	0.64	0.265
Mangrove	M09	Kochi	SW	0.998	0.889	1.17	0.24	0.6	0.22	0.57	0.47
Mangrove	M10	Panay	SW	0.998	0.955	1.16	0.9	0.99	0.94	0.98	1
Mangrove	M11	Can Gio	PW	0.532	0.18	0.748	0.47	0.8	0.5	0.85	NA
Mangrove	M11	Can Gio	SW	0.838	0.607	1.03	0.81	0.9	0.75	0.9	3.41
Mangrove	M12	Ho Cooc	SW	0.976	0.936	1.12	1.15	0.91	0.66	0.97	NA

Mangrove	M13	Ca Mau	SW	1	0.932	1.14	0.91	0.88	0.66	0.98	NA
Mangrove	M14	Bangrong	SW	1.05	0.791	1.19	1.12	0.96	0.79	0.83	3.2
Mangrove	M15	Badeldaob 2	PW	0.724	0.578	0.794	0.99	0.82	1.35	1	NA
Mangrove	M15	Badeldaob 2	SW	1.02	0.95	1.12	1.16	0.98	1.12	0.97	0.885
Mangrove	M16	Badeldaob 1	PW	0.784	0.765	0.853	0.97	1	0.97	1	NA
Mangrove	M16	Badeldaob 1	SW	0.948	0.904	1.25	0.64	0.82	0.61	0.78	0.834
Mangrove	M17	Sinnamary	SW	1.08	0.136	1.35	1.15	0.94	0.55	0.62	1.22
Mangrove	M18	Amazon	PW	1	0.301	1.14	0.1	0.02	0.54	0.37	NA
Mangrove	M18	Amazon	SW	1.03	0.761	1.11	0.95	0.97	0.95	0.97	4.63
Mangrove	M19	Paraiba	SW	0.745	0.684	1.25	0.63	0.66	0.04	0.1	0.32
Mangrove	M20	Paraty	PW	0.91	0.397	1	0.24	0.72	0.26	0.66	NA
Mangrove	M20	Paraty	SW	1.1	1.02	1.25	1.31	0.62	0.77	0.23	1.64
Mangrove	M21	Florianopolis	PW	0.632	0.55	0.753	0.67	0.95	0.77	0.96	NA
Mangrove	M21	Florianopolis	SW	1.04	0.654	1.22	0.42	0.41	0.33	0.35	0.78
Mangrove	M22	Guayas	SW	0.961	0.843	1.04	1.17	1	0.98	0.95	4.3
Mangrove	M23	Tana	PW	0.889	0.701	0.941	0.91	0.99	0.9	0.99	NA
Mangrove	M23	Tana	SW	0.991	0.894	1.16	1.03	0.96	0.96	0.99	NA
Mangrove	M24	Gazi Bay	SW	0.989	0.902	1.12	0.97	0.95	0.89	0.98	NA
Mangrove	M25	Nagada	SW	1.12	0.935	1.24	0.71	0.84	0.9	0.83	NA
Mangrove	M26	Ras Dege	SW	1.03	0.931	1.17	0.78	0.97	0.73	0.97	2.61
Mangrove	M27	Mtoni	SW	1.07	0.876	1.18	0.72	0.71	0.76	0.87	2.5
Mangrove	M28	Betsiboka	SW	1.09	0.927	1.23	1.15	0.96	0.75	0.82	NA
Mangrove	M29	Darwin	PW	1.36	0.675	1.57	0.95	0.67	1.2	0.87	NA
Mangrove	M29	Darwin	SW	1.11	0.959	1.2	0.19	0.25	0.12	0.1	4.21
Mangrove	M30	Johnstone	SW	1.03	0.955	1.08	1.21	0.96	0.62	0.35	NA
Mangrove	M31	Hinchinbrook	PW	0.958	0.873	1.04	0.92	0.98	0.92	0.97	NA
Mangrove	M31	Hinchinbrook	SW	1.03	0.879	1.08	-0.03	0.05	-0.05	0.12	1.87
Mangrove	M32	Burdekin	SW	0.993	0.95	1.13	0.83	0.98	0.84	0.99	NA
Mangrove	M33	Fitzroy	SW	1.07	0.985	1.09	0.92	1	0.84	0.99	NA
Mangrove	M34	1770	PW	0.89	0.475	0.983	1.05	0.97	0.68	0.84	NA
Mangrove	M34	1770	SW	1.13	1.01	1.19	0.64	0.69	0.13	0.05	2.49

Mangrove	M35	Jacobs Well	PW	0.955	0.921	1.09	0.87	0.84	0.85	0.8	NA
Mangrove	M35	Jacobs Well	SW	1.06	0.953	1.13	0.51	0.92	0.59	0.86	1.66
Mangrove	M36	Evans Head	PW	0.799	0.772	0.828	0.52	0.87	0.47	0.88	NA
Mangrove	M36	Evans Head	SW	1.07	0.56	1.53	1.1	0.92	0.57	0.44	1.26
Mangrove	M37	Newcastle	PW	1.12	0.963	1.25	1.21	0.98	1.21	0.99	NA
Mangrove	M37	Newcastle	SW	1.03	0.975	1.07	-0.11	0.14	-0.02	0.01	1.08
Mangrove	M39	Barwon Heads	PW	0.95	0.174	1.09	0.05	0.05	0.06	0.07	NA
Mangrove	M39	Barwon Heads	SW	1.11	1.07	1.12	1.07	0.94	1.09	0.91	1.18

Table S3 | TA and DIC outwelling rates from mangroves and saltmarshes, including method, location, season, and reference.

Ecosystem	Country	Site	Method	Latitude	Longitude	Season (tide)	TA outwelling (mmol/m^2/d)	DIC outwelling (mmol/m^2/d)	Reference
Saltmarsh	MA, USA	Sage Lot Pond	Eulerian	41.5546	-70.5071	spring		67	Tamborski, et al. [3]
Saltmarsh	MA, USA	Sage Lot Pond	Eulerian	41.5546	-70.5071	summer		358	Tamborski, et al. [3]
Saltmarsh	MA, USA	Sage Lot Pond	Eulerian	41.5546	-70.5071	fall		117	Tamborski, et al. [3]
Saltmarsh	MA, USA	Sage Lot Pond	Eulerian	41.5546	-70.5071	annual		180	Tamborski, et al. [3]
Saltmarsh	MA, USA	Sage Lot Pond	Eulerian	41.5546	-70.5071	spring		32	Wang, et al. [2]
Saltmarsh	MA, USA	Sage Lot Pond	Eulerian	41.5546	-70.5071	spring		88	Wang, et al. [2]
Saltmarsh	MA, USA	Sage Lot Pond	Eulerian	41.5546	-70.5071	summer		114	Wang, et al. [2]
Saltmarsh	MA, USA	Sage Lot Pond	Eulerian	41.5546	-70.5071	summer		118	Wang, et al. [2]
Saltmarsh	MA, USA	Sage Lot Pond	Eulerian	41.5546	-70.5071	summer		127	Wang, et al. [2]
Saltmarsh	MA, USA	Sage Lot Pond	Eulerian	41.5546	-70.5071	fall		147	Wang, et al. [2]
Saltmarsh	MA, USA	Sage Lot Pond	Eulerian	41.5546	-70.5071	fall		65	Wang, et al. [2]
Saltmarsh	MA, USA	Sage Lot Pond	Eulerian	41.5546	-70.5071	fall		118	Wang, et al. [2]
Saltmarsh	MA, USA	Sage Lot Pond	Eulerian	41.5546	-70.5071	winter		86	Wang, et al. [2]
Saltmarsh	MA, USA	Sage Lot Pond	Eulerian	41.5546	-70.5071	annual		95	Wang, et al. [2]
Saltmarsh	MA, USA	Sage Lot Pond	Eulerian	41.5546	-70.5071	summer		247	Chu, et al. [5]
Saltmarsh	MA, USA	Sage Lot Pond	Eulerian	41.5546	-70.5071	fall		170	Chu, et al. [5]
Saltmarsh	MA, USA	Sage Lot Pond	Eulerian	41.5546	-70.5071	average		171	
Saltmarsh	CA, USA	Suisun Wetland	Eulerian	38.1715	-122.0601	annual	21	22	Bogard, et al. [7]
Saltmarsh	VA, USA	Sweet Hall Marsh	Eulerian	37.5503	-76.8884	summer	63	82	Neubauer and Anderson [41]
Saltmarsh	VA, USA	Sweet Hall Marsh	Eulerian	37.5503	-76.8884	summer	58	66	Neubauer and Anderson [41]
Saltmarsh	VA, USA	Sweet Hall Marsh	Eulerian	37.5503	-76.8884	fall	26	33	Neubauer and Anderson [41]
Saltmarsh	VA, USA	Sweet Hall Marsh	Eulerian	37.5503	-76.8884	annual	36	44	Neubauer and Anderson [41]
Saltmarsh	NC, USA	Freeman Creek	Eulerian, resp. rates	34.5980	-77.3270	annual		52	Czapla, et al. [42]
Saltmarsh	SC, USA	Oyster Landing	Rn mass balance	33.3333	-79.2000	summer		40	Morris and Whiting [43]

Saltmarsh	GA, USA	Wassaw Sound	Lagrangian	31.9269	-80.9562	annual		62	Cai, et al. [44]
Saltmarsh	GA, USA	Duplin River	Lagrangian	31.4206	-81.2958	fall	90	120	Wang and Cai [9]
Saltmarsh	GA, USA	Duplin River	Lagrangian	31.4206	-81.2958	fall	53	78	Wang and Cai [9]
Saltmarsh	GA, USA	Duplin River	Lagrangian	31.4206	-81.2958	winter	21	40	Wang and Cai [9]
Saltmarsh	GA, USA	Duplin River	Lagrangian	31.4206	-81.2958	winter	5	38	Wang and Cai [9]
Saltmarsh	GA, USA	Duplin River	Lagrangian	31.4206	-81.2958	winter	3	35	Wang and Cai [9]
Saltmarsh	GA, USA	Duplin River	Lagrangian	31.4206	-81.2958	spring	109	130	Wang and Cai [9]
Saltmarsh	GA, USA	Duplin River	Lagrangian	31.4206	-81.2958	spring	29	89	Wang and Cai [9]
Saltmarsh	GA, USA	Duplin River	Lagrangian	31.4206	-81.2958	spring	10	33	Wang and Cai [9]
Saltmarsh	GA, USA	Duplin River	Lagrangian	31.4206	-81.2958	summer	18	68	Wang and Cai [9]
Saltmarsh	GA, USA	Duplin River	Lagrangian	31.4206	-81.2958	summer	34	62	Wang and Cai [9]
Saltmarsh	GA, USA	Duplin River	Lagrangian	31.4206	-81.2958	summer	51	69	Wang and Cai [9]
Saltmarsh	GA, USA	Duplin River	Lagrangian	31.4206	-81.2958	fall	34	40	Wang and Cai [9]
Saltmarsh	GA, USA	Duplin River	Lagrangian	31.4206	-81.2958	fall	50	75	Wang and Cai [9]
Saltmarsh	GA, USA	Duplin River	Lagrangian	31.4206	-81.2958	fall	41	68	Wang and Cai [9]
Saltmarsh	GA, USA	Duplin River	Lagrangian	31.4206	-81.2958	winter	27	73	Wang and Cai [9]
Saltmarsh	GA, USA	Duplin River	Lagrangian	31.4206	-81.2958	winter	-4	45	Wang and Cai [9]
Saltmarsh	GA, USA	Duplin River	Lagrangian	31.4206	-81.2958	winter	1	42	Wang and Cai [9]
Saltmarsh	GA, USA	Duplin River	Lagrangian	31.4206	-81.2958	spring	-22	12	Wang and Cai [9]
Saltmarsh	GA, USA	Duplin River	Lagrangian	31.4206	-81.2958	spring	21	42	Wang and Cai [9]
Saltmarsh	GA, USA	Duplin River	Lagrangian	31.4206	-81.2958	annual	11	36	Wang and Cai [9]
Saltmarsh	MA, USA	Duplin River	Eulerian	31.4458	-81.2857	winter		60	Wang, et al. [45]
Saltmarsh	MA, USA	Duplin River	Eulerian	31.4458	-81.2857	spring		35	Wang, et al. [45]
Saltmarsh	MA, USA	Duplin River	Eulerian	31.4458	-81.2857	spring		48	Wang, et al. [45]
Saltmarsh	MA, USA	Duplin River	Eulerian	31.4458	-81.2857	summer		10	Wang, et al. [45]
Saltmarsh	MA, USA	Duplin River	Eulerian	31.4458	-81.2857	fall		-44	Wang, et al. [45]
Saltmarsh	MA, USA	Duplin River		31.4458	-81.2857	average	30	53	
Saltmarsh	GA, USA	Altamaha River	Mixing model	31.3370	-81.3851	fall	69	82	Cai, et al. [46]
Saltmarsh	GA, USA	Satilla River	Mixing model	30.9726	-81.5087	fall	0	1	Cai, et al. [46]
Saltmarsh	China	Chuandong	Eulerian	33.0366	120.8407	fall	78	202	Yau, et al. [11]
Saltmarsh	China	Chuandong	Eulerian	33.0265	120.8991	summer (spring)		177	Chen, et al. [12]
Saltmarsh	China	Chuandong	Eulerian	33.0265	120.8991	summer (neap)		243	Chen, et al. [12]
Saltmarsh	China	Chuandong	Eulerian	33.0265	120.8991	winter (spring)		657	Chen, et al. [12]
Saltmarsh	China	Chuandong	Eulerian	33.0265	120.8991	winter (neap)		339	Chen, et al. [12]

Saltmarsh	China	Chuandong	Eulerian	33.0265	120.8991	annual		557	Chen, et al. [12]
Saltmarsh	China	Chuandong	Eulerian	33.0265	120.8991	average		363	
Saltmarsh	China	Chongming Dongtan	Eulerian	31.4808	122.0661	winter		1052	Liu, et al. [47]
Saltmarsh	China	Hangzuhou Bay	Eulerian	30.3548	121.1278	spring		1200	Zhu, et al. [48]
Saltmarsh	China	Zhangjiang Estuary	Darcy's Law	23.9206	117.4264	fall (spring)	-1	-1	Lu (unpublished)
Saltmarsh	China	Zhangjiang Estuary	Darcy's Law	23.9206	117.4264	fall (neap)	-4	-4	Lu (unpublished)
Saltmarsh	China	Zhangjiang Estuary	Darcy's Law	23.9206	117.4264	average	-2	-2	Lu (unpublished)
Saltmarsh	South Africa	Swartkops estuary	Eulerian	-33.8667	25.6333	annual		247	Winter, et al. [49]
Mangrove	FL, USA	Everglades	Lagrangian	25.3625	-81.0847	dry		21	Ho, et al. [50]
Mangrove	FL, USA	Everglades	Lagrangian	25.3625	-81.0847	dry		13	Ho, et al. [50]
Mangrove	FL, USA	Everglades	Eulerian	25.3625	-81.0847	dry	97	142	Reithmaier, et al. [14]
Mangrove	FL, USA	Everglades		25.3625	-81.0847	average	97	59	
Mangrove	Taiwan	Danshuei (*K. obovata*)	Conc. gradient	25.1372	121.4581	spring		-42	Li, et al. [51]
Mangrove	Taiwan	Danshuei (*K. obovata*)	Conc. gradient	25.1372	121.4581	summer		-17	Li, et al. [51]
Mangrove	Taiwan	Danshuei (*K. obovata*)	Conc. gradient	25.1372	121.4581	fall		-33	Li, et al. [51]
Mangrove	Taiwan	Danshuei (*K. obovata*)	Conc. gradient	25.1372	121.4581	annual		-43	Li, et al. [51]
Mangrove	Taiwan	Erhlin (*K. obovata*)	Conc. gradient	23.9342	120.3142	spring		708	Li, et al. [51]
Mangrove	Taiwan	Erhlin (*K. obovata*)	Conc. gradient	23.9342	120.3142	summer		242	Li, et al. [51]
Mangrove	Taiwan	Erhlin (*K. obovata*)	Conc. gradient	23.9342	120.3142	fall		-342	Li, et al. [51]
Mangrove	Taiwan	Erhlin (*K. obovata*)	Conc. gradient	23.9342	120.3142	winter		333	Li, et al. [51]
Mangrove	Taiwan	Erhlin (*K. obovata*)	Conc. gradient	23.9342	120.3142	annual		292	Li, et al. [51]
Mangrove	Taiwan	Erhlin (*K. obovata*)	Conc. gradient	23.9342	120.3142	spring		567	Li, et al. [51]
Mangrove	Taiwan	Erhlin (*K. obovata*)	Conc. gradient	23.9342	120.3142	summer		167	Li, et al. [51]
Mangrove	Taiwan	Erhlin (*K. obovata*)	Conc. gradient	23.9342	120.3142	fall		33	Li, et al. [51]
Mangrove	Taiwan	Erhlin (*K. obovata*)	Conc. gradient	23.9342	120.3142	winter		633	Li, et al. [51]
Mangrove	Taiwan	Erhlin (*K. obovata*)	Conc. gradient	23.9342	120.3142	annual		370	Li, et al. [51]
Mangrove	Taiwan	Erhlin Stream	Conc. gradient	23.9342	120.3142	average		344	Li, et al. [51]
Mangrove	Taiwan	Chiku (*A. marina*)	Conc. gradient	23.1186	120.0889	spring		33	Li, et al. [51]
Mangrove	Taiwan	Chiku (*A. marina*)	Conc. gradient	23.1186	120.0889	summer		292	Li, et al. [51]
Mangrove	Taiwan	Chiku (*A. marina*)	Conc. gradient	23.1186	120.0889	fall		117	Li, et al. [51]
Mangrove	Taiwan	Chiku (*A. marina*)	Conc. gradient	23.1186	120.0889	winter		33	Li, et al. [51]
Mangrove	Taiwan	Chiku (*A. marina*)	Conc. gradient	23.1186	120.0889	annual		121	Li, et al. [51]
Mangrove	Japan	Fukido River	Eulerian	24.4870	124.2304	dry		113	Ohtsuka, et al. [52]

Mangrove	Japan	Fukido River	Eulerian	24.4870	124.2304	wet		279	Ohtsuka, et al. [52]
Mangrove	Japan	Fukido River	Eulerian	24.4870	124.2304	average		196	Ohtsuka, et al. [52]
Mangrove	Japan	Iriomote Island	Eulerian	24.3838	123.8872	wet	44	11	Akhand, et al. [15]
Mangrove	China	Zhangjiang Estuary	Darcy's Law	23.9247	117.4219	dry (spring)	27	32	Lu (unpublished)
Mangrove	China	Zhangjiang Estuary	Darcy's Law	23.9247	117.4219	dry (neap)	23	25	Lu (unpublished)
Mangrove	China	Zhangjiang Estuary	Darcy's Law	23.9247	117.4219	average	25	29	Lu (unpublished)
Mangrove	India	Sundarbans	Eulerian	21.6612	88.3503	dry		202	Ray, et al. [16]
Mangrove	Philippines	Panay	Eulerian	11.8060	122.2020	dry		94	Ray, et al. [24]
Mangrove	Philippines	Panay	Eulerian	11.8060	122.2020	wet		164	Ray, et al. [24]
Mangrove	Philippines	Panay	Eulerian	11.8060	122.2020	annual		140	Ray, et al. [24]
Mangrove	Vietnam	Can Gio	Eulerian	10.5056	106.8825	dry (sym.)		352	Taillardat, et al. [25]
Mangrove	Vietnam	Can Gio	Eulerian	10.5056	106.8825	dry (sym.)		480	Taillardat, et al. [25]
Mangrove	Vietnam	Can Gio	Eulerian	10.5056	106.8825	dry (asym.)		678	Taillardat, et al. [25]
Mangrove	Vietnam	Can Gio	Eulerian	10.5056	106.8825	dry (interm.)		612	Taillardat, et al. [25]
Mangrove	Vietnam	Can Gio	Eulerian	10.5056	106.8825	dry (interm.)		339	Taillardat, et al. [25]
Mangrove	Vietnam	Can Gio	Eulerian	10.5056	106.8825	average		492	Taillardat, et al. [25]
Mangrove	Palau	Badeldaob 2	Eulerian	7.3911	134.5856	wet	2	10	Call, et al. [28]
Mangrove	Palau	Badeldaob 1	Eulerian	7.3667	134.5775	wet	48	79	Call, et al. [28]
Mangrove	French Guiana	Sinnamary	Eulerian	5.4500	-53.0111	dry		-75	Ray, et al. [29]
Mangrove	Brazil	Amazon	Eulerian	-0.8782	-46.6291	dry	15	20	Cabral, et al. [31]
Mangrove	Ecuador	Guayas	Eulerian	-2.5056	-79.8743	dry		6	Belliard, et al. [33]
Mangrove	Ecuador	Guayas	Eulerian	-2.5056	-79.8743	wet		22	Belliard, et al. [33]
Mangrove	Ecuador	Guayas	Eulerian	-2.5056	-79.8743	annual		17	Belliard, et al. [33]
Mangrove	Australia	Darwin	Eulerian	-12.5197	130.9060	dry	116	85	Sippo, et al. [38]
Mangrove	Australia	Karumba (living)	Lagrangian	-17.4265	140.8557	dry	951	1051	Sippo, et al. [53]
Mangrove	Australia	Karumba (dead)	Lagrangian	-17.4265	140.8557	dry	600	502	Sippo, et al. [53]
Mangrove	Australia	Hinchinbrook	Eulerian	-18.2440	146.2280	dry	21	22	Sippo, et al. [38]
Mangrove	Australia	1770	Eulerian	-24.1920	151.5698	dry	81	-97	Sippo, et al. [38]
Mangrove	Australia	Moreton Bay	Eulerian	-27.7775	153.4031	wet (spring)		183	Maher, et al. [54]
Mangrove	Australia	Moreton Bay	Eulerian	-27.7775	153.4031	wet (neap)		245	Maher, et al. [54]
Mangrove	Australia	Moreton Bay	Eulerian	-27.7775	153.4031	dry (spring)		340	Maher, et al. [54]
Mangrove	Australia	Moreton Bay	Eulerian	-27.7775	153.4031	annual		250	Maher, et al. [54]
Mangrove	Australia	Moreton Bay	Eulerian	-27.7775	153.4031	wet	96	212	Maher, et al. [55]

Mangrove	Australia	Moreton Bay	Eulerian	-27.7775	153.4031	average	96	246	
Mangrove	Australia	Jacobs Well	Eulerian	-27.7809	153.3796	wet/summer	12	83	Sippo, et al. [38]
Mangrove	Australia	Evans Head	Eulerian	-29.1208	153.4279	dry (22 mm)	60	61	Santos, et al. [40]
Mangrove	Australia	Evans Head	Eulerian	-29.1208	153.4279	wet (130 mm)	567	794	Santos, et al. [40]
Mangrove	Australia	Evans Head	Eulerian	-29.1208	153.4279	dry (28 mm)	90	86	Santos, et al. [40]
Mangrove	Australia	Evans Head	Eulerian	-29.1208	153.4279	dry (0 mm)	716	522	Santos, et al. [40]
Mangrove	Australia	Evans Head	Eulerian	-29.1208	153.4279	annual	358	358	Santos, et al. [40]
Mangrove	Australia	Newcastle	Eulerian	-32.8515	151.7675	wet	116	77	Sippo, et al. [38]
Mangrove	Australia	Western Port (WI)	Eulerian	-38.2361	145.2610	wet	310	460	Faber, et al. [56]
Mangrove	Australia	Western Port (WI)	Eulerian	-38.2361	145.2610	wet	110		Faber, et al. [56]
Mangrove	Australia	Western Port (WI)	Eulerian	-38.2361	145.2610	average	210	460	Faber, et al. [56]
Mangrove	Australia	Western Port (CI)	Eulerian	-38.2412	145.3167	wet	46	140	Faber, et al. [56]
Mangrove	Australia	Western Port (CI)	Eulerian	-38.2412	145.3167	wet	130		Faber, et al. [56]
Mangrove	Australia	Western Port (CI)	Eulerian	-38.2412	145.3167	average	88	140	Faber, et al. [56]
Mangrove	Australia	Barwon Heads	Eulerian	-38.2572	144.4870	wet	-1	-3	Sippo, et al. [38]

Table S4 | TA and DIC outwelling rates per site (averaged single observations from Table S3). Average annual temperature and average annual precipitation were gathered from corresponding publications or nearest weather stations. Tidal range, sediment accumulation rate (SAR), and carbon accumulation rate (CAR) were retrieved from global datasets[57].

Ecosystem	Country	Site	TA outwelling (mmol/m^2/d)	DIC outwelling (mmol/m^2/d)	Temperature (°C)	Precipitation (mm)	Tidal range (m)	SAR (mm/y)	CAR (gC/m^2/y)
Saltmarsh	MA, USA	Sage Lot Pond		171	10	530	1.2	2.9	118
Saltmarsh	CA, USA	Suisun Wetland	21	22	14	581	1.6	3.3	122
Saltmarsh	VA, USA	Sweet Hall Marsh	36	44	16	1158	0.2	5.6	115
Saltmarsh	NC, USA	Freeman Creek		52	17	1339	0.9	2.4	107
Saltmarsh	SC, USA	Oyster Landing		40	20	927	1.0	2.9	205
Saltmarsh	GA, USA	Wassaw Sound		62	19	1158	1.4	2.1	28
Saltmarsh	GA, USA	Duplin River	30	53	19	1158	1.4	2.1	28
Saltmarsh	GA, USA	Altamaha River	69	82	20	1114	1.4	3.4	145
Saltmarsh	GA, USA	Satilla River	0	1	21	1047	1.4	2.2	26
Saltmarsh	China	Chuandong		363	14	1100	3.1	40.0	392
Saltmarsh	China	Chongming Dongtan		1052	15	1022	4.1	15.0	18
Saltmarsh	China	Hangzuhou Bay		1200	17	1381	3.4	18.5	22
Saltmarsh	China	Zhangjiang Estuary	-2	-2	23	1679	2.6	14.2	405
Saltmarsh	South Africa	Swartkops estuary		247	19	563	1.4	5.0	80
Mangrove	FL, USA	Everglades	97	59	24	1534	2.2	2.7	176
Mangrove	Taiwan	Danshuei River		-43	21	2219	1.4	18.5	22
Mangrove	Taiwan	Erhlin Stream		344	23	1708	3.4	18.5	22
Mangrove	Taiwan	Chiku Stream		121	23	1708	0.9	18.5	22
Mangrove	Japan	Fukido River		196	24	2169	1.1	15.0	18
Mangrove	Japan	Iriomote Island	44	11	24	2342	1.1	15.0	18
Mangrove	China	Zhangjiang Estuary	25	29	23	1679	2.6	14.2	405
Mangrove	India	Sundarbans		202	28	1750	2.1	4.8	66
Mangrove	Philippines	Panay		140	26	2400	1.4	8.0	281
Mangrove	Vietnam	Can Gio		492	28	1350	2.9	10.0	228
Mangrove	Palau	Badeldaob 2	2	10	27	3700	1.1	2.6	158

Mangrove	Palau	Badeldaob 1	48	79	27	3700	1.1	2.2	70
Mangrove	French Guiana	Sinnamary		-75	28	1500	2.0	8.3	254
Mangrove	Brazil	Amazon	15	20	29	3000	5.3	8.3	254
Mangrove	Ecuador	Guayas		17	24	2321	2.0	5.8	462
Mangrove	Australia	Darwin	116	85	27	1694	5.3	6.3	168
Mangrove	Australia	Karumba	951	1051	34	820	0.9	8.5	294
Mangrove	Australia	Hinchinbrook	21	22	24	2001	2.2	2.7	143
Mangrove	Australia	1770	81	-97	22	1196	1.9	1.3	48
Mangrove	Australia	Moreton Bay	96	246	20	1478	1.2	5.8	150
Mangrove	Australia	Jacobs Well	12	83	20	1555	1.2	2.7	99
Mangrove	Australia	Evans Head	358	358	20	1500	1.1	4.2	74
Mangrove	Australia	Newcastle	116	77	18	1139	1.1	2.0	32
Mangrove	Australia	Western Port (WI)	210	460	19	810	1.4	1.3	21
Mangrove	Australia	Western Port (CI)	88	140	19	810	1.4	1.3	21
Mangrove	Australia	Barwon Heads	-1	-3	15	666	1.0	2.7	67

Table S5 | Net primary production (NPP) and major carbon fates of mangrove and saltmarsh production presented in MgC/ha/y as median ± SE (average). The unaccounted carbon fate was calculated as total NPP minus the sum of the major carbon fates. Using averages for DIC outwelling would close carbon budgets, as evident from slightly negative unaccounted carbon fates, but averages are unlikely to be representative of the skewed dataset with clear outliers. The direct CO_2 flux to the atmosphere and indirect production via DOC and POC respiration exceeds carbon burial in saltmarsh and mangrove sediments. The CO_2 return to the atmosphere is clearly important from a carbon budget perspective but does not contribute to carbon sequestration. Carbon burial and alkalinity outwelling are the two key pathways storing atmospheric CO_2, while CO_2 flux to the atmosphere is a recycling term linked to primary production and respiration.

**Parameter**	**Mangroves**	**Saltmarshes**	**Reference**
Aboveground NPP	5.1 ± 4.4 (5.2)	9.4 ± 1.1 (12.6)	Alongi [58]
Belowground NPP	9 ± 1.7 (13.2)	3.7 ± 0.3 (5)	Alongi [58]
Total NPP	14.1 ± 6.1 (18.4)	13.1 ± 1.4 (17.6)	Alongi [58]
CO2 outgassing water	2.2 ± 0.4 (3.4)	1.6 ± 0.3 (3)	Alongi [58]
Carbon burial	1.3 ± 0.2 (1.9)	1.3 ± 0.1 (1.7)	Wang, et al. [57]
POC outwelling	1.8 ± 0.2 (1.7)	0.3 ± 0.1 (0.6)	Alongi [58]
DOC outwelling	1.4 ± 2 (5.9)	1.3 ± 0.6 (2.6)	Alongi [58]
DIC outwelling	3.6 ± 2.1 (6.8)	2.5 ± 4.6 (10.6)	This study
Sum of carbon fates	10.2 (19.7)	7 (18.4)	
Unaccounted fate	3.9 (-1.3)	6.1 (-0.8)	

Table S6 | Global alkalinity balance of the ocean.

**Parameter**	**Tmol/y**	**Reference**
Riverine DIC	32	Middelburg, et al. [59]
Riverine PIC	21	Middelburg, et al. [59]
Submarine groundwater	1	Middelburg, et al. [59]
Submarine silicate	2.8	Middelburg, et al. [59]
Sulfur burial	4.7	Middelburg, et al. [59]
Denitrification	1.5	Middelburg, et al. [59]
Organic matter burial	3	Middelburg, et al. [59]
Tidal wetlands	4.6	This study
Total sources	*71*	
Open Ocean Carbonate Burial	23	Middelburg, et al. [59]
Ocean Margin Carbonate Burial	36	Middelburg, et al. [59]
Reverse Weathering	1	Middelburg, et al. [59]
Total sinks	*60*	

References

1. Egleston, E. S., Sabine, C. L. & Morel, F. M. Revelle revisited: Buffer factors that quantify the response of ocean chemistry to changes in DIC and alkalinity. *Global Biogeochem. Cycles* **24** (2010).
2. Wang, Z. A., Kroeger, K. D., Ganju, N. K., Gonneea, M. E. & Chu, S. N. Intertidal salt marshes as an important source of inorganic carbon to the coastal ocean. *Limnol. Oceanogr.* **61**, 1916-1931 (2016).
3. Tamborski, J. J., Eagle, M., Kurylyk, B. L., Kroeger, K. D., Wang, Z. A., Henderson, P. et al. Pore water exchange-driven inorganic carbon export from intertidal salt marshes. *Limnol. Oceanogr.* **66**, 1774-1792 (2021).
4. Song, S., Wang, Z. A., Gonneea, M. E., Kroeger, K. D., Chu, S. N., Li, D. et al. An important biogeochemical link between organic and inorganic carbon cycling: Effects of organic alkalinity on carbonate chemistry in coastal waters influenced by intertidal salt marshes. *Geochim. Cosmochim. Acta* **275**, 123-139 (2020).
5. Chu, S. N., Wang, Z. A., Gonneea, M. E., Kroeger, K. D. & Ganju, N. K. Deciphering the dynamics of inorganic carbon export from intertidal salt marshes using high-frequency measurements. *Mar. Chem.* **206**, 7-18 (2018).
6. Brooks, T., Kroeger, M., Mann, K., Wang, A., Ganju, Z., Suttles, N. O. K. et al. Geochemical data supporting investigation of solute and particle cycling and fluxes from two tidal wetlands on the south shore of Cape Cod, Massachusetts. *U.S. Geological Survey data release* **2012-19** (2021).
7. Bogard, M. J., Bergamaschi, B. A., Butman, D. E., Anderson, F., Knox, S. H. & Windham-Myers, L. Hydrologic export is a major component of coastal wetland carbon budgets. *Global Biogeochem. Cycles* **34**, e2019GB006430 (2020).
8. Correa, R. E., Xiao, K., Conrad, S. R., Wadnerkar, P. D., Wilson, A. M., Sanders, C. J. et al. Groundwater carbon exports exceed sediment carbon burial in a salt marsh. *Estuaries Coasts* **45**, 1-17 (2021).
9. Wang, Z. A. & Cai, W. J. Carbon dioxide degassing and inorganic carbon export from a marsh-dominated estuary (the Duplin River): A marsh CO_2 pump. *Limnol. Oceanogr.* **49**, 341-354 (2004).
10. Pérez-Lloréns, J., Brun, F., Andrıa, J. & Vergara, J. Seasonal and tidal variability of environmental carbon related physico-chemical variables and inorganic C acquisition in Gracilariopsis longissima and Enteromorpha intestinalis from Los Toruños salt marsh (Cádiz Bay, Spain). *J. Exp. Mar. Biol. Ecol.* **304**, 183-201 (2004).
11. Yau, Y. Y., Xin, P., Chen, X., Zhan, L., Call, M., Conrad, S. R. et al. Alkalinity export to the ocean is a major carbon sequestration mechanism in a macrotidal saltmarsh. *Limnol. Oceanogr.* **158–170**, 1-13 (2022).
12. Chen, X., Santos, I. R., Hu, D., Zhan, L., Zhang, Y., Zhao, Z. et al. Pore-water exchange flushes blue carbon from intertidal saltmarsh sediments into the sea. *Limnol. Oceanogr. Letters* **7**, 312-320 (2022).
13. Zhu, P., Chen, X., Zhang, Y., Zhang, Q., Wu, X., Zhao, H. et al. Porewater-derived blue carbon outwelling and greenhouse gas emissions in a subtropical multi-species saltmarsh. *Front. Mar. Sci.* **9**, 884951 (2022).
14. Reithmaier, G. M. S., Ho, D. T., Johnston, S. & Maher, D. T. Mangroves as a source of greenhouse gases to the atmosphere and alkalinity and dissolved carbon to the coastal ocean: A case study from the Everglades National Park, Florida. *J. Geophys. Res. Biogeosci.* **125**, e2020JG005812 (2020).
15. Akhand, A., Watanabe, K., Chanda, A., Tokoro, T., Chakraborty, K., Moki, H. et al. Lateral carbon fluxes and CO_2 evasion from a subtropical mangrove-seagrass-coral continuum. *Sci. Total Environ.* **752**, 142190 (2021).
16. Ray, R., Baum, A., Rixen, T., Gleixner, G. & Jana, T. Exportation of dissolved (inorganic and organic) and particulate carbon from mangroves and its implication to the carbon budget in the Indian Sundarbans. *Sci. Total Environ.* **621**, 535-547 (2018).

17. Akhand, A., Chanda, A., Manna, S., Das, S., Hazra, S., Roy, R. et al. A comparison of CO_2 dynamics and air-water fluxes in a river-dominated estuary and a mangrove-dominated marine estuary. *Geophys. Res. Lett.* **43**, 11,726-711,735 (2016).
18. Akhand, A., Chanda, A., Watanabe, K., Das, S., Tokoro, T., Chakraborty, K. et al. Low CO_2 evasion rate from the mangrove-surrounding waters of the Sundarbans. *Biogeochemistry* **153**, 95-114 (2021).
19. Akhand, A., Chanda, A., Watanabe, K., Das, S., Tokoro, T., Hazra, S. et al. Drivers of inorganic carbon dynamics and air–water CO_2 fluxes in two large tropical estuaries: Insights from coupled radon (^{222}Rn) and pCO_2 surveys. *Limnol. Oceanogr.* **9999**, 1-15 (2022).
20. Akhand, A., Chanda, A., Watanabe, K., Das, S., Tokoro, T., Hazra, S. et al. Reduction in riverine freshwater supply changes inorganic and organic carbon dynamics and air-water CO_2 fluxes in a tropical mangrove dominated estuary. *J. Geophys. Res. Biogeosci.* **126**, e2020JG006144 (2021).
21. Bouillon, S., Frankignoulle, M., Dehairs, F., Velimirov, B., Eiler, A., Abril, G. et al. Inorganic and organic carbon biogeochemistry in the Gautami Godavari estuary (Andhra Pradesh, India) during pre-monsoon: The local impact of extensive mangrove forests. *Global Biogeochem. Cycles* **17**, 1114 (2003).
22. Borges, A., Djenidi, S., Lacroix, G., Théate, J., Delille, B. & Frankignoulle, M. Atmospheric CO_2 flux from mangrove surrounding waters. *Geophys. Res. Lett.* **30**, 1558 (2003).
23. Linto, N., Barnes, J., Ramachandran, R., Divia, J., Ramachandran, P. & Upstill-Goddard, R. Carbon dioxide and methane emissions from mangrove-associated waters of the Andaman Islands, Bay of Bengal. *Estuaries Coasts* **37**, 381-398 (2014).
24. Ray, R., Miyajima, T., Watanabe, A., Yoshikai, M., Ferrera, C. M., Orizar, I. et al. Dissolved and particulate carbon export from a tropical mangrove-dominated riverine system. *Limnol. Oceanogr.* **66**, 3944-3962 (2021).
25. Taillardat, P., Willemsen, P., Marchand, C., Friess, D., Widory, D., Baudron, P. et al. Assessing the contribution of porewater discharge in carbon export and CO_2 evasion in a mangrove tidal creek (Can Gio, Vietnam). *J. Hydrol.* **563**, 303-318 (2018).
26. Taillardat, P., Ziegler, A. D., Friess, D. A., Widory, D., Van, V. T., David, F. et al. Carbon dynamics and inconstant porewater input in a mangrove tidal creek over contrasting seasons and tidal amplitudes. *Geochim. Cosmochim. Acta* **237**, 32–48 (2018).
27. Borges, A. V., Abril, G. & Bouillon, S. Carbon dynamics and CO_2 and CH_4 outgassing in the Mekong delta. *Biogeosciences* **15**, 1093-1114 (2018).
28. Call, M., Sanders, C. J., Macklin, P. A., Santos, I. R. & Maher, D. T. Carbon outwelling and emissions from two contrasting mangrove creeks during the monsoon storm season in Palau, Micronesia. *Estuar. Coast. Shelf Sci.* **218**, 340-348 (2019).
29. Ray, R., Gérard, T., Romain, W., Vincent, V., Gerd, G., Sylvain, M. et al. Mangrove-derived organic and inorganic carbon exchanges between the Sinnamary estuarine system (French Guiana, South America) and the Atlantic Ocean. *J. Geophys. Res. Biogeosci.* **125**, e2020JG005739 (2020).
30. Ray, R., Michaud, E., Aller, R., Vantrepotte, V., Gleixner, G., Walcker, R. et al. The sources and distribution of carbon (DOC, POC, DIC) in a mangrove dominated estuary (French Guiana, South America). *Biogeochemistry* **138**, 297-321 (2018).
31. Cabral, A., Dittmar, T., Call, M., Scholten, J., de Rezende, C. E., Asp, N. et al. Carbon and alkalinity outwelling across the groundwater-creek-shelf continuum off Amazonian mangroves. *Limnol. Oceanogr. Letters* **6**, 369-378 (2021).
32. Cotovicz Jr, L. C., Vidal, L. O., de Rezende, C. E., Bernardes, M. C., Knoppers, B. A., Sobrinho, R. L. et al. Carbon dioxide sources and sinks in the delta of the Paraíba do Sul River (Southeastern Brazil) modulated by carbonate thermodynamics, gas exchange and ecosystem metabolism during estuarine mixing. *Mar. Chem.* **226**, 103869 (2020).
33. Belliard, J.-P., Hernandez, S., Temmerman, S., Suello, R. H., Dominguez-Granda, L. E., Rosado-Moncayo, A. M. et al. Carbon dynamics and CO_2 and CH_4 exchange in the mangrove dominated Guayas river delta, Ecuador. *Estuar. Coast. Shelf Sci.* **267**, 107766 (2022).
34. Bouillon, S., Dehairs, F., Schiettecatte, L.-S. & Borges, A. V. Biogeochemistry of the Tana estuary and delta (northern Kenya). *Limnol. Oceanogr.* **52**, 46-59 (2007).

35. Bouillon, S., Dehairs, F., Velimirov, B., Abril, G. & Borges, A. V. Dynamics of organic and inorganic carbon across contiguous mangrove and seagrass systems (Gazi Bay, Kenya). *J. Geophys. Res.* **112**, G02018 (2007).
36. Bouillon, S., Middelburg, J. J., Dehairs, F., Borges, A. V., Abril, G., Flindt, M. R. et al. Importance of intertidal sediment processes and porewater exchange on the water column biogeochemistry in a pristine mangrove creek (Ras Dege, Tanzania). *Biogeosciences* **4**, 311-322 (2007).
37. Ralison, O. H., Borges, A. V., Dehairs, F., Middelburg, J. & Bouillon, S. Carbon biogeochemistry of the Betsiboka estuary (north-western Madagascar). *Org. Geochem.* **39**, 1649-1658 (2008).
38. Sippo, J. Z., Maher, D. T., Tait, D. R., Holloway, C. & Santos, I. R. Are mangroves drivers or buffers of coastal acidification? Insights from alkalinity and dissolved inorganic carbon export estimates across a latitudinal transect. *Global Biogeochem. Cycles* **30**, 753-766 (2016).
39. Rosentreter, J. A. & Eyre, B. D. Uncertainties about the role of terrestrial dissolved inorganic carbon and alkalinity loads in buffering the Great Barrier Reef lagoon against ocean acidification. *Authorea* (2024).
40. Santos, I. R., Maher, D. T., Larkin, R., Webb, J. R. & Sanders, C. J. Carbon outwelling and outgassing vs. burial in an estuarine tidal creek surrounded by mangrove and saltmarsh wetlands. *Limnol. Oceanogr.* **64**, 996-1013 (2019).
41. Neubauer, S. C. & Anderson, I. C. Transport of dissolved inorganic carbon from a tidal freshwater marsh to the York River estuary. *Limnol. Oceanogr.* **48**, 299-307 (2003).
42. Czapla, K. M., Anderson, I. C. & Currin, C. A. Net ecosystem carbon balance in a North Carolina, USA, salt marsh. *J. Geophys. Res. Biogeosci.* **125**, e2019JG005509 (2020).
43. Morris, J. T. & Whiting, G. J. Emission of gaseous carbon dioxide from salt-marsh sediments and its relation to other carbon losses. *Estuaries* **9**, 9-19 (1986).
44. Cai, W. J., Wang, Z. A. & Wang, Y. The role of marsh-dominated heterotrophic continental margins in transport of CO_2 between the atmosphere, the land-sea interface and the ocean. *Geophys. Res. Lett.* **30** (2003).
45. Wang, S. R., Di Iorio, D., Cai, W. J. & Hopkinson, C. S. Inorganic carbon and oxygen dynamics in a marsh-dominated estuary. *Limnol. Oceanogr.* **63**, 47-71 (2018).
46. Cai, W.-J., Wang, Y. & Hodson, R. E. Acid-base properties of dissolved organic matter in the estuarine waters of Georgia, USA. *Geochim. Cosmochim. Acta* **62**, 473-483 (1998).
47. Liu, J., Yu, X., Chen, X., Du, J. & Zhang, F. Utility of radium quartet for evaluating porewater-derived carbon to a saltmarsh nearshore water: implications for blue carbon export. *Sci. Total Environ.* **764**, 144238 (2021).
48. Zhu, P., Chen, X., Zhang, Y., Zhang, Q., Wu, X., Zhao, H. et al. Porewater-derived blue carbon outwelling and greenhouse gas emissions in a subtropical multi-species saltmarsh. *Front. Mar. Sci.* **9**, 621 (2022).
49. Winter, P. E., Schlacherl, T. A. & Baird, D. Carbon flux between an estuary and the ocean: a case for outwelling. *Hydrobiologia* **337**, 123-132 (1996).
50. Ho, D. T., Ferrón, S., Engel, V. C., Anderson, W. T., Swart, P. K., Price, R. M. et al. Dissolved carbon biogeochemistry and export in mangrove-dominated rivers of the Florida Everglades. *Biogeosciences* **14**, 2543-2559 (2017).
51. Li, S. B., Chen, P. H., Huang, J. S., Hsueh, M. L., Hsieh, L. Y., Lee, C. L. et al. Factors regulating carbon sinks in mangrove ecosystems. *Global Change Biol.* **24**, 4195-4210 (2018).
52. Ohtsuka, T., Onishi, T., Yoshitake, S., Tomotsune, M., Kida, M., Iimura, Y. et al. Lateral export of dissolved inorganic and organic carbon from a small mangrove estuary with tidal fluctuation. *Forests* **11**, 1041 (2020).
53. Sippo, J. Z., Maher, D. T., Schulz, K. G., Sanders, C. J., McMahon, A., Tucker, J. et al. Carbon outwelling across the shelf following a massive mangrove dieback in Australia: Insights from radium isotopes. *Geochim. Cosmochim. Acta* **253**, 142–158 (2019).
54. Maher, D. T., Santos, I. R., Golsby-Smith, L., Gleeson, J. & Eyre, B. D. Groundwater-derived dissolved inorganic and organic carbon exports from a mangrove tidal creek: The missing mangrove carbon sink? *Limnol. Oceanogr.* **58**, 475-488 (2013).

55. Maher, D. T., Call, M., Santos, I. R. & Sanders, C. J. Beyond burial: Lateral exchange is a significant atmospheric carbon sink in mangrove forests. *Biol. Lett.* **14**, 20180200 (2018).
56. Faber, P. A., Evrard, V., Woodland, R. J., Cartwright, I. C. & Cook, P. L. Pore-water exchange driven by tidal pumping causes alkalinity export in two intertidal inlets. *Limnol. Oceanogr.* **59**, 1749-1763 (2014).
57. Wang, F., Sanders, C. J., Santos, I. R., Tang, J., Schuerch, M., Kirwan, M. L. et al. Global blue carbon accumulation in tidal wetlands increases with climate change. *Natl. Sci. Rev.* **8**, nwaa296 (2021).
58. Alongi, D. M. Carbon cycling in the world's mangrove ecosystems revisited: Significance of non-steady state diagenesis and subsurface linkages between the forest floor and the coastal ocean. *Forests* **11**, 977 (2020).
59. Middelburg, J. J., Soetaert, K. & Hagens, M. Ocean alkalinity, buffering and biogeochemical processes. *Rev. Geophys.* **58**, e2019RG000681 (2020).
